# Assessing the mental health needs of Yazidi adolescents and young adults in an Iraqi Kurdi IDP Camp: a focus group study

**DOI:** 10.1186/s12939-024-02182-8

**Published:** 2024-05-01

**Authors:** Roberto Benoni, Chiara Giacomelli, Giuditta Vegro, Faroq Hamo, Riccardo Avesani, Pietro Albi, Michela Gatta, Francesca Moretti

**Affiliations:** 1 Department of Diagnostics and Public Health, University of Verona, Strada Le Grazie, 8, Verona, 37134 Italy; 2 RedLab - Darkroom over the Borders, Verona, Italy; 3 Imprint of Hope, Erbil, Iraq; 4 Department of Women’s and Children’s Health, Padua University Hospital, Padua, Italy; 5 Department of Neurosciences, Biomedicine and Movement Sciences, University of Verona, Verona, Italy

## Abstract

**Background:**

Internally displaced people (IDP) in Iraq are 1.2 million (as March 2023). Protracted refugee status endangers the mental health, especially of minorities who survived persecution and conflict, such as the Yazidis. This study aims to identify the mental health needs of Yazidi adolescents and young adults (AYA) in the IDP camp of Bajed Kandala (Iraqi Kurdistan).

**Methods:**

A focus group discussion (FGD) study was conducted between April and August 2022. The FGDs involved AYAs, as well as the staff of the clinic of the Bajed Kandala camp. An inductive approach was adopted referring to the ’theme’ as the unit of content analysis of the text. All FGDs were recorded and transcribed. The analysis was carried out independently by two researchers. The inter-rater agreement was assessed through the Cohen’s k.

**Results:**

A total of 6 FGDs were conducted. The participants were 34 of whom 21 (61.8%) females with a median age of 18.5 years (IQR 17.0–21.0). A total of 156 themes were found as relevant to the objective of this study. Four main areas and twelve subareas of needs in mental health were identified. The interrater agreement over the main area and subareas was good (κ = 0.78 [0.95CI 0.69–0.88], κ = 0.82 [0.95CI 0.73–0.91], respectively). The four areas had a similar frequency: Activities (28.2%), Individual (27.6%), Social relationships (22.4%) and Places/setting (21.8%). The subareas ‘community’ and ‘internal resources’ were labelled as negative 85.7% and 61.9% of the time, respectively. These sub-areas referred to stigma and self-stigma towards mental health. The subarea ‘female condition’ was always considered as negative, as well as the subareas ‘camp’ and ‘tent’ referring to housing as an important social determinant of mental health.

**Conclusions:**

Community stigma and self-stigma are two still important factors preventing the achievement of mental well-being. Alongside these, a gender gap in mental health was identified in the FGDs. These factors should be taken into account in order to guide future mental health interventions in refugee camps.

**Supplementary Information:**

The online version contains supplementary material available at 10.1186/s12939-024-02182-8.

## Background

At the end of 2022, the Internal Displacement Monitoring Centre reported 62,479,565 refugees worldwide [1]. This number has doubled over 20 years as a result of conflict, persecution or events that seriously disturb public order. Mostly are internally displaced persons (IDP) who have been living for years in refugee camps or other regions of their country far from home. The humanitarian situation in Iraq is particularly dramatic. As of March 2023, nearly 1.2 million Iraqis continue to be IDP and 70% have been displaced for more than five years [2]. Among the IDP in Iraq, the majority lives in Iraqi Kurdistan and 21.4% reside in the governorate of Dohuk, which currently hosts 15 camps, of which 11 are tented [3]. The population is predominantly Yazidi, an ethno-religious minority that has suffered persecution, discrimination, and other abuses for centuries and a genocide perpetrated by ISIS in 2014. Their religion has been exploited by Islamic State of Iraq and the Levant (ISIL) to perpetrate mass killings, forced conversions, child abductions and the sexual enslavement of thousands of women [4]. Before the invasion by the ISIL, the Yazidi community was estimated at between 300,000 and 550,000-700,000 members. As of August 2020, an estimated 3,000 Yazidis are still missing or captive [4].

The psychosocial problems and needs of the displaced population are extremely high; an increasing number of suicides, suicide attempts and other self-injuries behaviors have been observed among the IDP [5]. It has been recognized that many refugees suffer from psychosocial distress as a consequence of violence, war, continued displacement, and the economic and social hardships they have experienced in the past and continue to experience in the present [5]. Among the Yazidis, fear is still widespread: 91% of IDPs indicated a perception of greater safety in the camps compared to the external living environment [6]. The violence suffered by the Yazidi community in 2014 resulted in significant psychological disorders among the younger population at the time of the attacks (6–18 years old), including sleep disorders (71%), depression (36.8%), adjustment (21.8%) and post-traumatic stress disorders (10.5%) [7]. It has been reported that lack of education further increases the risk for children and young people to be exposed to psychosocial distress, difficulties in communication, relationships, self-expression, and self-awareness [8]. The risk of mental health problems disproportionately affects women in two distinct phases: firstly, during the conflict due to gender-based violence, and subsequently after resettlement, stemming from the gender disparity in access to services and education [9]. This led to a significantly higher prevalence of MH disorder, such as depression and PTSD, among women [10]. A high prevalence of mental disorders was also found in other countries with a similar background. In Afghanistan, it was reported that 44% of the sample surveyed had experienced more than four traumatic events in the last ten years, 68% had experienced some form of depression, and 72% and 42% showed symptoms of anxiety and post-traumatic stress disorder, respectively [11]. 

While primary care programs are often accompanied by psychological and social care interventions, this individual and collective trauma, regardless of cultural and ethnic backgrounds, requires medical and mental health care that can offer a sense of security, stability and orientation: unfortunately, the level of support is often insufficient and inadequate given the overwhelming and urgent needs people face in camp life [12]. Psychosocial support interventions should be culturally oriented not only to respect cultural differences, but also to achieve a better outcome. In fact, mental health is particularly sensitive to the cultural substrate, so a medical anthropology-oriented approach is necessary to achieve good results [13]. Integrating mental health care into the health interventions of governmental and non-governmental organizations (NGOs), and providing educational activities and opportunities, is crucial to ensure a better quality of life for refugees and IDPs. Several gaps in the provision of MH and psychosocial support services to Yazidi population have been reported in the literature, including the lack of MH professional staff, limited MH facilities and services offered by both governments and NGOs, and insufficient support (budget, recruitment, and encouragement) for mental health assistance. Among these should be noted the lack or insufficiency of MH specialized services informed by the real needs of the population [14]. Furthermore, a lack of studies and data on MH of specific at-risk groups, such as adolescents, and minority groups, such as Yazidis, has been reported [15].Therefore, the main aim of this study was to identify the mental health needs of Yazidi adolescents and young adults (AYA) in the IDP camp of Bajed Kandala in Iraqi Kurdistan. The second objective was to explore the factors influencing help seeking behavior for mental health and to identify opportunities for actions in the camp.

## Methods

### Ethical approval

The research was performed following the ethical standards of the 1964 Declaration of Helsinki and the ethical approval was provided on 13 April 2023 by the Bajed Kandala camp management comprising Barzani Charity Foundation (BCF) and United Nations High Commissioner for Refugees (UNHCR). All participants in this study provided written informed consent.

### Study design, setting and population

This is a qualitative study using focus group discussions (FGDs). It was carried out in the Bajed Kandala camp, one of the most densely populated in the Duhok region, in northern Iraq. It was opened in August 2013 as a transit site for Syrian refugees but since August 2014 it has been used to host IDPs, Iraqi Kurds, the majority of whom belongs to the Yazidi population [16]. It has an estimated population of 8470 people, 32% of whom aged between 6 and 17 years, with a similar sex distribution (females represent 47% of this age group) [3]. Even though 91% of the displaced noted the precariousness of the shelters (accommodating an average of 5.6 people each), 96% of the camp population said they would like to stay, and the remaining 4% have no ideas about possible relocation. The camp is divided into two areas (BK1 and BK2) and in each of them there is a health facility run by NGOs that provide primary health care services, but without a specific mental health one.

All people aged between 14 and 24 years living in Bajed Kandala camp and people working in the BK2 health facility were considered eligible for FGD. Those unable to give informed consent or without legal representative were excluded.

### Sample size

According to the available literature, a sample of six focus groups is considered sufficient to achieve a saturation of 90% of the possible topics expressed in the focus group discussion [17]. Therefore, six FGDs were organized and included in the analysis with a minimum of five persons per group. People were enrolled in a non-probabilistic method, by convention, asking progressively those who met the inclusion criteria and accepted to participate.

### Data collection

Six FGDs were conducted between April and August 2022. Five FGDs were conducted among Yazidi internally displaced people, of which 2 homogeneous by sex and 3 heterogeneous. One FGD was performed with health facility staff of BK2 to investigate their perceptions of AYAs’ needs. This distribution was used to promote self-expression in the homogeneous groups and to stimulate discussion in the heterogeneous ones by maximizing the production of topics [18, 19].

All FGDs were conducted in local language (Kurmanji) with a cultural mediator fluent in both Kurmanji and English (FH) leading the groups, while a second member of the research team (RB), expert in FGDs methodology, supervised the process to ensure proper data collection and validity of results. The FGDs were conducted following a guiding grid of questions and topics to be covered (Annex 1). The grid used an indirect questioning approach, starting from simple questions regarding everyday life experience to gradually get into the main topic of mental health. A medical doctor was available during and after the FGD to give psychological first aid, for participants who may get nervous or aroused because of the topics covered during the discussion. In addition, a debriefing session to discourse on how one felt following the discussion was conducted at the end of each FGD.

All the FGDs were recorded and transcribed. All the text transcriptions were then translated and analysed in English. Before starting the FGDs, a brief sociodemographic questionnaire was administered to each of the participants (Annex 2).

### Content analysis

An inductive content analysis was conducted. In the present study we selected as unit of analysis (i.e the basic unit of text that is classified during content analysis) any part of the text expressing a concept related to the main aim of the study (”Theme”). Themes can be expressed as single words, phrases, sentences, and paragraphs [20].

The following rules were applied to identify the themes emerged in the FGDs and codified them: (1) read the text repeatedly to obtain the full overview of the text; (2) highlight the parts of the text that appear to capture a theme of interest (e.g., phrases that refer to mental health, needs, barriers, psychological difficulties, etc.…); (3) reread the FGDs transcription and take notes of the content to which the primary themes that have been highlighted refer (coding of the themes); (4) Group the themes that refer to similar concepts into categories and assign a preliminary label; (5) repeat Steps 3 and 4 until all highlighted themes are grouped into categories; (6) organize the retrieved categories into a hierarchical structure (identifying areas and eventual subarea) based on the relationship between categories; (7) develop definitions and codes (label) for each area and subarea. Categories were defined respecting the need to maximize mutual exclusivity [21].

Three researchers (RB, PA and FM) read through two FGD in order to identify possible themes and their relationship. All potential classification were discussed to obtain a consensus. A classification index code was created for the subsequent independent analysis of FGDs (Supplementary material). Two raters (CG and GV) independently assessed and coded the FGDs. They assigned every theme to categories, area and subareas and gave them a positive or negative values in regard the potential impact on mental health. Discrepancies in the labeling process were discussed and resolved by involving a third rater (RB).

### Statistical analysis

A descriptive analysis was first conducted; frequency rates and percentages were used for categorical variables and medians with interquartile range for continuous variables.

To identify the issues that were discussed more frequently by the participants, a frequency count of all areas and subareas retrieved in the 6 FGDs was performed. To explore the participants‘ needs, a bivariate frequency distribution of the positive/negative values given by the participants for each subarea was constructed. Proportions for categorical variables were compared by the χ2 or Fisher’s exact test. Continuous variables were compared via Mann-Whitney-U non-parametric test.

The “Cohen’s k” was calculated to assess the agreement between the two raters for both the areas and the sub-areas. The “Cohen’s k” was considered satisfying for values between 0.61 and 1.00 (from moderate to almost perfect level of agreement) [22].

A *p*-value < 0.05 was considered significant. All analyses were performed using the R software (version 4.1.1).

## Results

Six FGDs were conducted. A total of 34 people attended it, 27 Yazidi AYAs, with a median age of 17.0 years (IQR 16.0–19.0), and 7 clinic staff (FGD number 4). Median age of AYAs wasn’t significantly different between males and females (*p* = 0.267). The composition of each FGDs is shown in Table 1.

**Table 1:** Characteristics of participants distinguished by focus group discussion (FGD). FGD 4 was conducted with the clinic staff of BK2 while the others FGDs with Yazidi refugee adolescents and young adults

	FGD 1 (*n* = 6)	FGD 2 (*n* = 5)	FGD 3 (*n* = 5)	FGD 4 (*n* = 7)	FGD 5 (*n* = 6)	FGD 6 (*n* = 5)	Overall (*n* = 34)
**Sex**							
Female	6 (100%)	0 (0,0%)	3 (60,0%)	3 (42,9%)	5 (83,3%)	4 (80,0%)	21 (61,8%)
Male	0 (0,0%)	5 (100%)	2 (40,0%)	4 (57,1%)	1 (16,7%)	1 (20,0%)	13 (38,2%)
**Age (years)**							
Median (IQR)	20 (18–20)	17 (17–19)	17 (17–19)	29 (24–30)	17 (16–19)	14 (14–16)	19 (17–21)
**Education level**							
Elementary	3 (50,0%)	0 (0,0%)	0 (0,0%)	0 (0,0%)	0 (0,0%)	0 (0,0%)	3 (8,8%)
High school	3 (50,0%)	5 (100%)	5 (100%)	0 (0,0%)	5 (83,3%)	2 (40,0%)	20 (58,8%)
Middle school	0 (0,0%)	0 (0,0%)	0 (0,0%)	1 (14,3%)	1 (16,7%)	3 (60,0%)	5 (14,7%)
University	0 (0,0%)	0 (0,0%)	0 (0,0%)	6 (85,7%)	0 (0,0%)	0 (0,0%)	6 (17,6%)
**School attendance**							
No	3 (50,0%)	0 (0,0%)	1 (20,0%)	7 (100%)	0 (0,0%)	0 (0,0%)	11 (32,4%)
Yes	3 (50,0%)	5 (100%)	4 (80,0%)	0 (0,0%)	6 (100%)	5 (100%)	23 (67,6%)

**Fig. 1:**
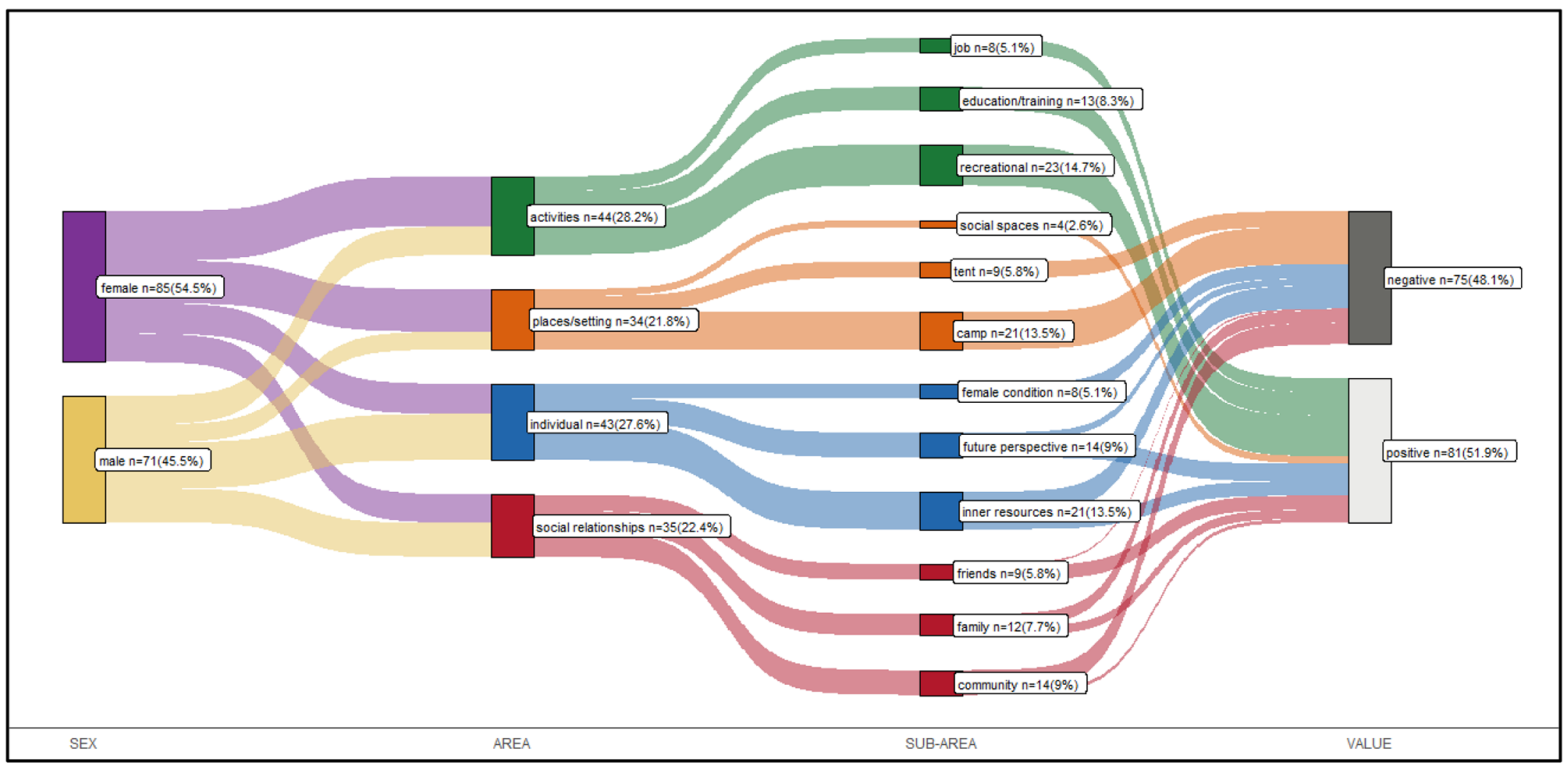
Sankey diagram of the theme identified in the content analysis distinguished by sex, area, subarea, and value (positive/negative)

A total of 156 themes were found as relevant to the objective of this study. Four main areas of needs in mental health of AYA refugees were identified: “Activities”, “Individual”, “Social relationships” and “Places/setting”. The interrater agreement over the main areas was good (alpha = 0.78 [0.95CI 0.69–0.88]. The sub-areas identified were 12 (Fig. 1). The interrater agreement over the sub-areas was also good (alpha = 0.82 [0.95CI 0.73–0.91]).

### Activities

The impact of the activities on the mental health of AYA refugees was retrieved in 44 (28.2%) themes. All themes were labelled with a *positive* value. The types of activities that were identified as sub-areas were “Recreational”, “Education and training” and “Job”.

“Recreational” was retrieved in 23 (52.3%) themes and emerged more frequently among AYAs (*n* = 22/36, 61.1%) compared to the staff of the health facility (*n* = 1/8, 12.5%, *p* = 0.027, Table 2). This type of activity was mainly related to the idea of breaking a routine that creates a sense of immobility and stressed their protracted refugee situations:

- *“If nothing changes, everything becomes boring, with the same routine.” Young boy.*
- *“I feel good [doing these activities], every day is the same routine.” Young boy.*
- *“I want to do something different from [the/my] usual routine.” Young boy.*

Doing recreational activities was also linked to the concept of not thinking to their situation and problems:

- *“Most of the activities allow me not to think about things that can make me feel bad”.* Young girl.
- *“When I have a picnic and forget. The change in routine.” Young boy*.
- “*If I am busy with these activities I do not think about other things and my situation*.” Young girl.

“Education and training” and “Job” were identified in 13 (29.5%) and 8 (18.2%) sentences, respectively. These two activities were more frequently reported by the health facility’s staff, together accounting for the 87.5% of the sub-area themes in the “Activities” area, compared to AYAs (38.9%, *p* = 0.027).

The activities in these two subareas were also often linked to the “not-thinking” issue:

- *“Probably the opportunity to work or to have an income is one way, but also providing activities, such as tailoring courses for women, or language or software courses for everyone, these things can help take your mind off your problems.”* Male health facility staff.
- *“Those who work, those who have a business, those who have job opportunities, don’t have a lot of time to think, don’t spend too much time in tents, so if they don’t think so much about themselves and their condition, they will not have psychological problems.”* Male health facility staff.

These types of activities were also often linked to having an income and to gain economic independence for good mental health as well. This concept was often linked to the household rather than the individual:

- *“Teach them handicrafts to start a job or a small business to help themselves and their families get some money so they will be fine.”* Male health facility staff.
- *“For me the really important thing is to find a job, to try to help my father.” Young boy.*
- *“I want English and Information Technology (IT) courses because each has its own importance: the English is important for the college; the computer is important for college and job.” Young girl.*

**Table 2:** Number and percentages of the area e subarea identified in the focus group discussion distinguished by type of participants: adolescent and young adults (AYA) or clinic staff

	Yazidi AYAs (*n* = 127)	Clinic staff (*n* = 29)	Overall (*n* = 156)
**Activities**	**36 (28,3%)**	**8 (27,6%)**	**44 (28,2%)**
Job	5 (3,9%)	3 (10,3%)	8 (5,1%)
Recreational	22 (17,3%)	1 (3,4%)	23 (14,7%)
Education and training	9 (7,1%)	4 (13,8%)	13 (8,3%)
**Individual**	**36 (28,3%)**	**7 (24,1%)**	**43 (27,6%)**
Female condition	4 (3,1%)	4 (13,8%)	8 (5,1%)
Future perspective	12 (9,4%)	2 (6,9%)	14 (9,0%)
Inner resources	20 (15,7%)	1 (3,4%)	21 (13,5%)
**Social relationships**	**31 (24,4%)**	**4 (13,8%)**	**35 (22,4%)**
Friends	9 (7,1%)	0 (0,0%)	9 (5,8%)
Community	13 (10,2%)	1 (3,4%)	14 (9,0%)
Family	9 (7,1%)	3 (10,3%)	12 (7,7%)
**Place and settings**	**24 (18,9%)**	**10 (34,5%)**	**34 (21,8%)**
Camp	16 (12,6%)	5 (17,2%)	21 (13,5%)
Social spaces	2 (1,6%)	2 (6,9%)	4 (2,6%)
Tent	6 (4,7%)	3 (10,3%)	9 (5,8%)

### Individual

Individual needs and the person’s own role in mental health were the second most frequent area with 43 (27.6%) themes referring to this. The “Individual” area accounted for the 36.6% of all themes retrieved in male, while it represented only the 20% in women (*p* = 0.019). Of all themes in this area, 25 (58.2%) were labelled as *negative*.

The three subareas identified were “Inner resources”, “Future perspective”, and “Female condition”.

“Inner resources” was the most frequent sub-area (*n* = 21, 48.8%) and was labelled as negative in 13 (61.9%). Positive value was expressed through personal capability and the chance to communicate and talk about their problems and through a positive attitude towards possible change:

- *“I try to find hope for myself by talking about the good things and avoiding the bad”*. Young girl.
- *“The important thing is having the possibility to communicate”*. Young boy.
- *“It is important to remember the problem. It makes us strong. It is important to remember the solutions to the problem.”* Young boy.

On the contrary, the negative value was found in the personal attitude of problem removal and in not being able to talk to others. It was also shaped by the lack of trust in clinical help and in other people in the camp:

- *“If a friend of mine gets isolated because he has a problem, I would go and talk to him to try to make him forget the problem.”* Young boy.
- *“I never tried to talk about my problems with anyone.”* Young boy.
- *“I believe that these are our problems, and we can solve them by ourselves. We don’t need them [psychologists].” Young boy*.
- *“There are people with psychological problems who do not express their feelings because there is no one they trust”.* Male health facility staff.
- *“We cannot do anything; we have to stay silent. There are very few people that we could trust, we cannot trust anyone.”* Young girl.

In the area “Individual”, the sub-area “Future perspective” was most frequently labelled as positive (*n* = 10/18, 55.6%, *p* = 0.003). The positive future perspective was associated with the idea of leaving the camp and going to another country, mainly in Europe. It was seen as a hope that makes them feel good and as the possibility of achieving their dream:

- *“There was a time when I studied another language, like German or English, because I would like to go to Germany.”* Young girl.
- *“When I finish high school, I want to go abroad, to Holland, where my brother lives.”* Young boy.
- *“Many people can build on their dreams abroad. I think it can be a first step.”* Young boy.
- *“Any family would want to go to Europe, to a safe area and be peaceful even in their inner feelings.”* Male clinic staff.

The “Female condition” was reported 8 times (18.6%) and always as negative (*p* = 0.003). Health facility staff (*n* = 4/7, 57.1%) reported it more frequently than AYAs (*n* = 4/36, 11.1%, *p* = 0.019, Table 2). This was linked to the observation that all suicides within the camp were committed by girls and their prolonged periods spent in the tents:

- *“I think that maybe, because they are girls, the concept of suicide is seen differently”.* Young girl.
- *“At the same time, even among women, they tend to isolate themselves and remain enclosed in tents”*. Male health facility staff.
- *“But if we open a place for them [women], if the community sees one of the girls there, they would not accept it.”* Male health facility staff.
- *“All seven suicides were of girls.”* Female health facility staff.

### Social relationships

The area of social relationships and their impact on a person’s mental health emerged in 35 (22.4%) themes. A negative value was given to 20 (57.1%) themes. Three main sub-area were found: “Community”, “Family” and “Friends”.

The themes related to “Community” (*n* = 14, 40.0%) were more often identified as negative (*n* = 12/20, 60.0%) than the other sub-areas. (*p* = 0.002). In this sub-area, the stigma surrounding people with mental health needs and the sentiment that there is no one to talk freely about one’s problems and who can understand them were found:

- *“They are afraid that people will think they are mad.”* Young boy.
- *“People are ridiculed. If a person is ridiculed, he may think about committing suicide.”* Young boy.
- *“They are tired of not getting the thing they want. [.] they need somebody that understand[s] them, but they don’t have somebody to talk [to]. They want to say [speak] but they don’t have a person to understand them and believe and listen to them.”* Young girl.

The subarea “Family” was retrieved in 12 (34.3%) themes of the “Social relation” area, with similar frequency of both values, positive (*n* = 5, 41.7%) and negative (*n* = 7, 58.3%). Considering the negative value, the family member were sometimes considered as a source of concern, such as for health issues, or might be a cause of conflict:

- *“So, it is not only to take our minds off our situation but also to help our own families.”* Young girl.
- *“My mother suffers from diabetes. So, we need money. […] Now my dad is sick and if we have no [money] I think things will be bad.”* Young girl.
- *“Sometimes my ideas are different from those of my family and having this conflict is one of the problems.”* young boy.

On the other hand, the family was also seen as a focal point and support that complements the sub-area “Friends” (*n* = 9, 25.7%, positive = 8, 88.9%) to cope with problems and improve mental well-being:

- *“For me, well-being is being with my family and spending time with them.”* Young girl.
- *“Their families would encourage them to come [to the psychological support service].”* Male health facility staff.
- “O*therwise, we met our friends and family. These are the most important for mental wellbeing. Sometimes we express our feeling[s], we need someone to express, like our friends or someone else, because they are having the same problems, so we need someone else to talk to*.” Young girl.
- *“I can’t have the solution alone, so I share the problem with my friends and look for a solution with them.”* Young boy.

### Places and setting

This area was found in 34 (21.8%) themes and was mostly reported by females (*n* = 24, 28.2%, male = 10, 14.1%). It was labelled mainly as negative (*n* = 31, 91.1%, *p* < 0.001).

The “Camp” (*n* = 21, 61.8%) and the “Tent” (*n* = 9, 26.5%) sub-areas were the main element negatively impacting the mental health of Yazidi AYAs. They were all labelled as negative. Their impact was related to not having a chance to fulfil themselves, to having to stay locked in the camp or tent:

- “*I think mental well-being depends on so many different things, physical, mental, social, economic, contextual, and sexual. So, we have to focus on these things. Since we live in a refugee camp most of us have a problem with one of these aspects.*” Male health facility staff.
- *“In camps there are no place[s] to study and to study outside the camp is hard, we have only one tent, and inside there is no electricity. So, it’s hard.”* Young girl.
- *“These are the activities we prefer because they are the ones that allow us not to have to stay in tents and this is the most important thing for our well-being.”* Young female.
- *“If they have no activities […] they don’t spend too much time in the tents, […] whereas those who have nothing to do and stay closed in the tents are fine.”* Male health facility staff.

In contrast, the ‘Social spaces’ sub-area (*n* = 4, 11.8%) was always rated positively as a place where people can interact and spend time outside the tents:

- *“They opened clubs for youth and adolescents, and they do a bit of everything, […]. This would help the young people in the camp.”* Male health facility staff.

## Discussion

The purpose of this study was to identify the mental health needs of Yazidi AYAs in an IDP camp in northwestern Iraqi Kurdistan. Four main areas have been identified, with a substantially even distribution: Activities (28.2%), Individual (27.6%), Social relationships (22.4%) and Place and Settings (21.8%).

Adolescence is a critical time of life stage with a continuum of physical, cognitive, behavioral, and psychosocial changes that is characterized by increasing levels of individual autonomy, a growing sense of identity, self-esteem, and progressive independence from adults [23]. This period of intense change can physiologically constitute a fertile ground for the development of psychic suffering. Refugees, especially adolescents exposed to and experiencing war, are even more at risk of developing psychological problems [24]. Previous study showed how children and adolescents living in refugee camps urgently need psychosocial support [25].

**Fig. 2:**
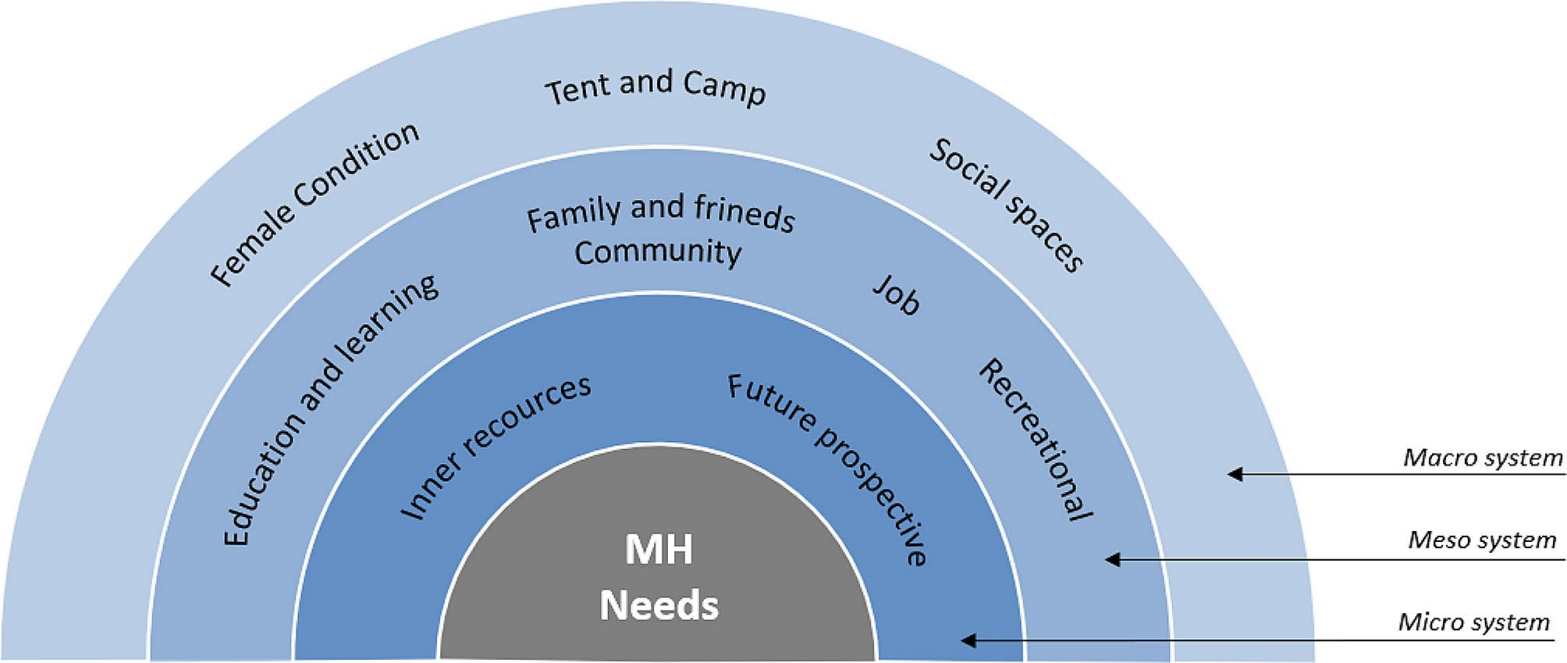
Subarea of mental health (MH) needs identified in the focus group discussions analysis stratified according to the Bronfenbrenner’s Ecological Systems Theory

The four main areas identified could be divided according to the Bronfenbrenner’s Ecological models of human development (Fig. 2) in a continuum from microsystem (Individual) to mesosystem (Social relationships and Activities) to macrosystem (Places and setting, and the subarea Female condition) [26, 27].

Starting from the macrosystem, an important subarea was about the female condition that is related to the fact all suicides in the camp were committed by girls and the fact that they are spending a lot of time isolated in their tents. For this reason, this subarea has always been labelled with a negative value in relation to mental health. According to a previous study, Yazidi women who survived war atrocities represent a highly traumatized population, their perceived social rejection seems to play a role in the relationship between trauma exposure and mental health [28]. Interestingly, this need for better mental health support for females as an at-risk category emerged most clearly from the clinic staff (57.1%), whereas the AYAs did not perceive this difference (11.1%). This is an important aspect on which to raise awareness amongst AYAs of both genders as in this age group most health seeking behaviors are directed towards physical problems (mainly infectious diseases) and only in adult females’ mental health emerged as a reason for clinical consultation [29].

Tents emerged as an isolating element and a negative factor for mental health in all the Yazidi AYAs, which impacts across all ecological systems. It was mainly reported by females and was mostly labelled as negative amongst all the themes in the Places and Setting area. Living in the tent and in the camp negatively impacts the mental health of Yazidi AYAs, which relates to not having a chance to fulfil themselves and to having to stay locked in the camp or tent. These results are consistent with the available literature. Braun-Lewensohnand Al-Sayed highlighted that “more time passes, and the adolescents see no change, but rather continue to live at the refugee camp for a long period of time with no other options, they become less resilient, their resources (human, social, and cultural capital are depleted), and they are also less cognitively hopeful” [30].

The testimonies collected from Yazidi people from BK camp confirm that the condition of living in the camp is a factor adversely affecting their individual resources and their health at different levels (physical, mental, sexual and social). Housing is a well-known social determinant of health for both communicable and non-communicable diseases. Poor shelter conditions in refugee camps have been associated with increasing mental distress and depression [31]. Psychological home defined by Sigmon and colleagues as “a sense of belonging in which self-identity is tied to a particular place” is the aspect most at risk in protracted refugee situations and has one of the greatest impacts on mental well-being [32].

Another concerning factor found in the mesosystem is the community stigma for people with mental health needs and the idea that there is no one to talk freely about one’s problems and who can understand them. The Community subarea was indeed significantly more often labelled as negative compared to the other subarea of Social relationships. The community has an important role in mental health and our findings underline the difficulties for people to trust each other and to express their emotional inner-world. Stigma negatively impacts health seeking behavior for mental health care [33]. Based on previous studies Iraqis have often been reluctant to seek treatment for mental health fearing family and social marginalization [34]. In this setting it is therefore important to implement interventions to reduce both self-stigma and internalized stigma among people with mental health disorders and stigma among families and caregivers. It has been shown to improve access to care and clinical outcomes [35].

The family was seen as a source of controversial feelings (positive = 41.7%, negative = 58.3%). The negative value was given to the fact that family members are a cause of concern for AYAs, who feel obliged to them. The strain resulting from refugee status has a negative effect on family relationships and the parental network, which can lead to internal family tensions and increased social isolation with a consequent negative impact on mental health [36]. Alongside this, there are other underlying biological, cultural and economic factors that can negatively impact the mental health of AYAs and their families as refugee war-survivors (i.e., low parental engagement, poor family communication and silence about war-related experiences) [37].

On the other hand, the family is seen as a focal and supportive point for sharing and coping with problems. The family support network also includes, with positive value, friends who are always considered as a reference for mental wellbeing needs.

Strong parental and social support, as well as personal and community resources, are some important factors that facilitate the ability to overcome the difficult situations and transitions faced by these adolescents, which aid in reducing negative emotional and behavioral outcomes [38]. The scoping review on social support by Wachter and colleagues found that out of 41 included studies, 23 (57%) and 19 (46%) indicated family and friends as the main source of social support for resettled refugees [39]. The family also has the role of protecting and relaying a sense of identity and cultural heritage after being eradicated from one’s home [36]. For Kurdish refugees, recreating cultural practices at the family level has proven to help them cope with migration-related losses, strengthening their collective identity and restoring hope for the future [40].

In the mesosystem, the area Activities played the main role as positive factors and emerged as the most frequent need for mental well-being of AYA refugees. The role attributed to activities is mainly that of breaking the routine and of the possibility to use time that would otherwise be flat. This is an important compass to guide future actions in the camp, and this finding underlines the need for activity and self-determination [27]. Artistic activities, especially those which involve active participation, for example participatory photography, may provide tools to address the holistic wellbeing of adolescent refugees in camps [41]. Both art therapy and sport have proven to be an effective tool to reduce mental distress and improve the mental health of refugees in protracted situations, particularly for those suffering from post-traumatic stress disorder and depression, conditions that can lead to suicidal ideation and attempts [42, 43].

In the microsystem (Individual area), future perspectives subarea emerged as an important topic and a key support for mental well-being. Kaethe Weingarten theorized the idea of reasonable hope as a focal point for supporting refugee people despite uncertainty, disappointment, and apparent hopelessness. It is defined as “the process of making sense of what exists now in the belief that this prepares us to meet what lies ahead. With reasonable hope, the present is filled with working not waiting” [44].

The subarea Inner resources highlighted the importance of expressing their feelings, to have somebody to communicate with. Indeed, it was labelled as negative (61.9%) identifying themes in which issues of incommunicability emerged and the fact that the mental health problem was a personal affair that could not be shared, but also as positive (38.1%) from themes about the importance of sharing individual problems with others for mental well-being. These individual barriers to sharing are often determined by self-stigma and self-judgement in seeking help [45]. Furthermore, the Individual area shows a gender gap, being less frequent in females than males. This could be due to the impact of conforming to traditional masculinity standards on the way men should deal with mental health problems and seek help [46]. On the other hand, women, and girls face risks specific to their refugee status such as health complications, particularly for pregnant women, physical harm and injuries, and risks of exploitation and gender-based violence. This has a dreadful negative impact on their mental well-being and on their view of future prospects through the lens of reasonable hope. Future intervention should therefore prioritize awareness and sensitization of AYAs regarding mental health and, at the same time, provide activities and workshops to improve the individual in their personal value. Secondly, it is important to work with local clinical staff to ensure a culturally oriented psychological support service for the Yazidi population that allows people to find a safe place to talk about themselves and to create a dedicated place for women to share their experiences and activities, emphasizing the importance of gender-matching interventions [47].

This study has some limitations. First, there are inherent limitations to FGDs, such as the tendency for certain types of socially acceptable opinions to emerge and for certain participants to dominate the discussion process. However, the FGDs conduction helped to ensure fluent discussion and active contributions by all participants. To limit social desirability bias, some FGDs were formed homogeneously by sex, which was important given age and cultural background. In addition, the main topic was explored through an indirect questioning approach, and total confidentiality of the discussion was remembered throughout. Secondly, although the number of six FGDs is considered sufficient to have a saturation of topics a larger number of FGDs (particularly for clinical staff) would have allowed for a more representative sample. It would also have been useful to conduct key informant interviews to have individual data especially by healthcare provider and NGO staff. Finally, the study is monocentric and based only on the situation in a single refugee camp. Despite these limitations, the results help shed light on the main determinants of young refugees’ mental health, providing guidance on which improvements and interventions may be effectively implemented.

## Conclusions

Exploring the complex interplay of factors affecting the mental well-being of Yazidi AYA refugees is essential for providing valuable insights into mental health needs. Indeed, this explorative study allowed to identify the unique challenges faced by young refugees, particularly in protracted camp situations. The impact of the camp environment, including living conditions in tents and the isolation experienced by many, emerged as a significant factor negatively affecting mental health. Moreover, there is still a long way to go to combat stigma, especially at the community level but also at the family level. Furthermore, it emerged how self-stigma also represents a barrier to seeking adequate support. Lastly, it was found that being involved in activities and housing as a social determinant of health are two perceived fundamental aspects for maintaining a good mental health. It is also crucial to consider gender-specific needs, as evidenced by the unique challenges faced by young women within the camp environment.

These findings emphasize the need for targeted interventions and support services aimed at addressing the mental health needs of Yazidi AYA refugees. Such interventions should prioritize activities that promote social interaction and skill-building, while also addressing stigma and promoting open communication about mental health.

### Electronic supplementary material

Below is the link to the electronic supplementary material.

Supplementary Material 1

## Data Availability

The datasets generated and/or analysed during the current study are available from the corresponding author on reasonable request.

## References

[1] The United Nation High Commissioner for Refugees (UNHCR). Refugee Data Finder. Availabe at https://www.unhcr.org/refugee-statistics/download/?url=3kMw [Last accessed 21/07/23].

[2] United Nations High Commissioner for Refugees (UNHCR). Iraq refugee crisis. https://www.unrefugees.org/emergencies/iraq/ [last accessed 21/07/2023].

[3] Global Camp Coordination and Camp Management (CCCM) Cluster. Iraq IDP Camp Profiling. Round XVI. 2022. https://repository.impact-initiatives.org/document/reach/d1e02a4b/REACH_IRQ_Factsheet_CampProfiling_IRQ1705_Jan2023_v4.pdf [last accessed 21/07/2023].

[4] European Union Agency for Asylum (EUAA). Religious and ethnic minorities, and stateless persons. Yazidi. 2021. https://euaa.europa.eu/country-guidance-iraq-2021/2152-yazidi [last accessed 21/07/2023].

[5] Haroz EE, Decker E, Lee C. Evidence for suicide prevention and response programs with refugees: A systematic review and recommendations. Geneva: United Nations High Commissioner for Refugees. 2018. https://www.unhcr.org/en-au/5e15d3d84.pdf [Last accessed 21/07/23].

[6] International Organization for Migration (IOM). Understanding Ethno-Religious Groups in Iraq: Displacement and Return report. 2019. https://reliefweb.int/report/iraq/understanding-ethno-religious-groups-iraq-displacement-and-return-report [last accessed 21/07/2023].

[7] Ceri V, Özlü-Erkilic Z, Özer Ü, Yalcin M, Popow C, Akkaya-Kalayci T (2016). Psychiatric symptoms and disorders among yazidi children and adolescents immediately after forced migration following ISIS attacks. Neuropsychiatr.

[8] The United Nation High Commissioner for Refugees (UNHCR). COI Note on the Situation of Yazidi IDPs in the Kurdistan Region of Iraq. 2019. https://www.refworld.org/pdfid/5cd156657.pdf [Last accessed 21/07/23].

[9] Bendavid E, Boerma T, Akseer N, Langer A, Malembaka EB, Okiro EA, Wise PH, Heft-Neal S, Black RE, Bhutta ZA, BRANCH Consortium Steering Committee (2021). The effects of armed conflict on the health of women and children. Lancet.

[10] Ahmed DR, Heun R (2023). The prevalence of psychiatric disorders among yazidi people results from ISIS invasion and consecutive trauma: a systematic review. Asian J Psychiatr.

[11] Sayed GD. Mental Health in Afghanistan: Burden, Challenges and the Way Forward. World Bank, 2011. https://documents1.worldbank.org/curated/en/692201467992810759/pdf/658840WP00PUBL0736B0MHinAfghanistan.pdf [Last accessed 16/04/24].

[12] Kizilhan JI, Noll-Hussong M (2017). Individual, collective, and transgenerational traumatization in the Yazidi. BMC Med.

[13] Rice AN, Harris SC. Issues of cultural competence in mental health care. J Am Pharm Assoc (2003). 2021 Jan-Feb;61(1):e65-e68. 10.1016/j.japh.2020.10.015. Epub 2020 Nov 5. PMID: 33160868. 10.1016/j.japh.2020.10.015 33160868

[14] Ahmed DR. Assessment of Mental Health and Psychosocial Support Limitations, Needs, and Recommendations in Iraq. Intervention 20(2):p 193–194, Jul–Dec. 2022. 10.4103/intv.intv_13_22.

[15] Ahmed DR (2022). Mental health problems in Iraq: a systematic review. Glob Psychiatry Arch.

[16] Office for the Coordination of Humanitarian Affairs (OCHA). Bajid Kandala Camp Profile, Duhok: Iraq Internal Displacement Crisis. November 2014. 2015. https://www.humanitarianresponse.info/en/operations/iraq/document/bajid-kandala-camp-profile-duhok-iraq-internal-displacement-crisis-november [last accessed 21/07/23].

[17] Guest G, Namey E, McKenna K (2017). How many focus groups are Enough? Building an evidence base for nonprobability sample sizes. Field Methods.

[18] Grønkjær M, Curtis T, de Crespigny C, Delmar C (2011). Analysing group interaction in focus group research: impact on content and the role of the moderator. Qualitative Stud.

[19] Halcomb EJ, Gholizadeh L, DiGiacomo M, Phillips J, Davidson PM. Literature review: considerations in undertaking focus group research with culturally and linguistically diverse groups. J Clin Nurs. 2007;16(6):1000-11. 10.1111/j.1365-2702.2006.01760.x. PMID: 17518876. 10.1111/j.1365-2702.2006.01760.x 17518876

[20] Weber RP (1990). Basic Content Analysis.

[21] Hsieh HF, Shannon SE (2005). Three approaches to qualitative content analysis. Qual Health Res.

[22] McHugh ML (2012). Interrater reliability: the kappa statistic. Biochem Med (Zagreb).

[23] Mitchell K. Adolescent sexual and reproductive health a toolkit for humanitarian setting, a companion to the Inter-agency Field Manual on Reproductive Health in Humanitarian Settings. Save the children and UNFPA; 2009.

[24] Bean T, Derluyn I, Eurelings-Bontekoe E, Broekaert E, Spinhoven P (2007). Comparing psychological distress, traumatic stress reactions, and experiences of unaccompanied refugee minors with experiences of adolescents accompanied by parents. J Nerv Ment Dis.

[25] Ceri V, Özlü-Erkilic Z, Özer Ü, Yalcin M, Popow C, Akkaya-Kalayci T (2016). Psychiatric symptoms and disorders among yazidi children and adolescents immediately after forced migration following ISIS attacks. Neuropsychiatrie.

[26] Bronfenbrenner U (1994). Ecological models of human development. Int Encyclopedia Educ.

[27] Drumm RD, Pittman SW, Perry S (2003). Social work interventions in refugee camps: an ecosystems approach. J Social Service Res.

[28] Ibrahim H, Ertl V, Catani C, Ismail AA, Neuner F (2018). Trauma and perceived social rejection among yazidi women and girls who survived enslavement and genocide. BMC Med.

[29] Cetorelli V, Burnham G, Shabila N (2017). Health needs and care seeking behaviours of Yazidis and other minority groups displaced by ISIS into the Kurdistan Region of Iraq. PLoS ONE.

[30] Braun-Lewensohn O, Al-Sayed K (2018). Syrian adolescent refugees: how do they cope during their stay in refugee camps?. Front Psychol.

[31] Conzatti A, Kershaw T, Copping A, Coley D (2022). A review of the impact of shelter design on the health of displaced populations. Int J Humanitarian Action.

[32] Sigmon ST, Whitcomb SR, Snyder CR, Fisher AT, Sonn CC, Bishop BJ (2002). Psychological home. Psychological sense of community.

[33] Schnyder N, Panczak R, rowth N, Schultze-Lutter F. Association between mental health-related stigma and active help-seeking: systematic review and meta-analysis. Br J Psychiatry. 2017;210(4):261–268. 10.1192/bjp.bp.116.189464. Epub 2017 Feb 2. Erratum in: Br J Psychiatry. 2017 Sep;211(3):184. PMID: 28153928. 10.1192/bjp.bp.116.189464 28153928

[34] Böge K, Hahn E, Strasser J, Schweininger S, Bajbouj M, Karnouk C (2022). Psychotherapy in the Kurdistan region of Iraq (KRI): preferences and expectations of the kurdish host community, internally displaced- and Syrian refugee community. Int J Soc Psychiatry.

[35] Waqas A, Malik S, Fida A, Abbas N, Mian N, Miryala S, Amray AN, Shah Z, Naveed S (2020). Interventions to reduce Stigma related to Mental Illnesses in Educational Institutes: a systematic review. Psychiatr Q.

[36] Bunn M, Zolman N, Polutnik Smith C, Khanna D, Hanneke R, Betancourt TS, Weine S. Family-based mental health interventions for refugees across the migration continuum: a systematic review. SSM-Mental Health. 2022;100153. 10.1016/j.ssmmh.2022.100153. 10.1016/j.ssmmh.2022.100153 PMC10392776 37529116

[37] Sangalang CC, Vang C (2017). Intergenerational trauma in Refugee families: a systematic review. J Immigr Minor Health.

[38] Braun-Lewensohn O (2015). Coping and social support in children exposed to mass trauma. Curr Psychiatry Rep.

[39] Wachter K, Bunn M, Schuster RC, Boateng GO, Cameli K, Johnson-Agbakwu CE (2021). A Scoping Review of Social Support Research among refugees in Resettlement: implications for conceptual and empirical research. J Refug Stud.

[40] Kevers R, Rober P, De Haene L (2017). The role of collective identifications in family processes of post-trauma reconstruction: anexploratory study with kurdish refugee families and their diasporic community. Kurd Stud.

[41] Andemicael A (2011). Positive energy: a review of the role of artistic activities in refugee camps.

[42] Rowe C, Watson-Ormond R, English L, Rubesin H, Marshall A, Linton K, Amolegbe A, Agnew-Brune C, Eng E (2017). Evaluating art therapy to heal the effects of Trauma among Refugee Youth: the Burma Art Therapy Program evaluation. Health Promot Pract.

[43] Gerber M, ollege F, de Quervain D, Filippou K, Havas E, Knappe F, Ludyga S, Meier M, Morres ID, Panagos A, Pühse U, Ramadan K, Seelig H, Theodorakis Y, von Känel R, Hatzigeorgiadis A (2021). Effects of an exercise and sport intervention among refugees living in a Greek refugee camp on mental health, physical fitness and cardiovascular risk markers: study protocol for the SALEEM pragmatic randomized controlled trial. Trials.

[44] Weingarten K. Reasonable hope: construct, clinical applications, and supports. Fam Process. 2010;49(1):5–25. 10.1111/j.1545-5300.2010.01305.x. PMID: 20377632. 10.1111/j.1545-5300.2010.01305.x 20377632

[45] Bär J, Pabst A, Röhr S, Luppa M, Renner A, Nagl M, Dams J, Grochtdreis T, Kersting A, König HH, Riedel-Heller SG (2021). Mental Health Self-Stigma of Syrian refugees with Posttraumatic stress symptoms: investigating Sociodemographic and Psychopathological correlates. Front Psychiatry.

[46] Seidler ZE, Dawes AJ, Rice SM, Oliffe JL, Dhillon HM (2016). The role of masculinity in men’s help-seeking for depression: a systematic review. Clin Psychol Rev.

[47] Ward J, Marsh M. (2006) Sexual violence against women and girls in war and its aftermath: realities, responses and required resources. Symposium on Sexual Violence in Conflict and Beyond. http://www.svri.org/sites/default/files/attachments/2016-01-15/CCEF504C15AB277E852571AB0071F7CE-UNFPA.pdf [last accessed 11/07/2023].

