# Supplementary material for: Assessing the mental health needs of Yazidi adolescents and young adults in an Iraqi Kurdi IDP Camp: a focus group study
Source: Int J Equity Health. 2024 May 1;23:88. doi: 10.1186/s12939-024-02182-8 (PMC11064332; doi:10.1186/s12939-024-02182-8)
Supplement: Supplementary file 1 — Supplementary Material 1 [file 12939_2024_2182_MOESM1_ESM.docx]

**SUPPLEMENTARY MATERIAL**

**Classification index code for focus group discussion analysis**:

**Individual**: by individual we mean inner, personal and/or individual condition related to mental health:

We defined it as:

- Inner resources: Characteristics of personality referring to cognitive, emotional, experiential competencies, and attitudes in relation to mental health; especially, referring to self-determination and resilience.
- Future perspective: The impact on mental health of how an individual thinks about the future, anticipates future consequences, and plans for it (i.e., how I feel about future perspective? What hope do I have towards them?)
- Female condition: the cultural, social, and personal impact that being female in the camp has on mental health. What are their specific risk factors (i.e., more time in the tent spent alone)

**Activities**: by activities we defined all practical, physical, or intellectual behaviours and/or things that give the opportunity to move within the camp and that in participants' statements have significant relevance to mental health.

We defined it as:

- All the activity during their daily routine or extra activities with an impact on the mental health and well-being of the person.
- positive or negative values linked with daily routine and with extra activities.
- We divided it in three subareas according to the type of activity reported (Recreational, i.e., sport; Educational and training; Job).

**Place and settings**: by place and settings we mean how the living conditions and the housing situation affect their mental health.

We defined it as:

- Condition in the camp and impact of living in a tent.
- Gaps, shortages, or absence of services in the refugee camp.

**Relationship**: by relationship we mean how social ties are related to the topic of mental health, in particular the three social ties emerged are Community, Family, and Friends.

We defined it as:

- Could they express how they feel in the community/ in the family/ with friends?
- Positive/negative values about community thinking/ about family thinking/ about friends thinking.
- How family/community/friends could contribute to their well-being.

Annex 1 – Question guide for the focus group discussion (FGD).

| **Main Objective:**  To identify the mental health needs of Yazidi adolescents and young adults (AYA) in the refugee camp of Bajed Kandala in Iraqi Kurdistan |
| --- |
| **Topics to explore in the debate: Through the debate between participants get information on:**   - Vision/means of health and mental health for the participant. - Mental health vision/means for the community. - Needs in mental health and related services. |
| **Focus Group Steps:**  **1. Introduction** *(approximately 10 minutes)*  The facilitator presents the overview and the objectives of the discussion. Participants introduce themselves**.**  **2.** **Understanding-building phase** *(approximately 10 minutes)*  To start the discussion and the relationship between the group, the facilitator asks simple questions to the participants.  **3. Deep Discussion** *(60-90 minutes)*  The facilitator asks questions related to the main purpose of the focus group, which encourages discussion that reveals the thoughts and opinions of the participants. It is at this stage that the most important information is collected.  **4. Conclusion** *(approximately 10 minutes)*  The facilitator summarizes the information or conclusions discussed and participants clarify or confirm the information. The facilitator answers any questions, thanks the participants and indicates the next steps. |
| **Questions:**   1. What do you do during the day? what are your daily activities? 2. How do you feel during these activities? 3. Have you ever heard about mental health? 4. What do you mean by mental health and what is its importance? 5. What does mental health mean for your community (parents, family, friends, etc.)? 6. What do you do when you don't feel well psychologically/mentally (sad, stressed, worried, angry, aggressive)? 7. In the community, how do you treat people with mental/psychological problems? What about you, what do you think? 8. What do you think needs to improve you mental wellbeing? |
| **Role of the Session Facilitator/Moderator:**   - Inform participants about the objectives of the session. - Request authorization for audio recording. - Ensure the anonymity and confidentiality of the data. - Explain the rules of the group session: speak one at a time; avoid arguments; no one should dominate the discussion; importance of everyone's participation - Put the main question for debate - Encourage everyone's participation by asking small open-ended questions - Encourage debate in order to deepen the issues to be explored - Intervene if any participant deviates from the topic at hand - Summarize the discussion that has already taken place - Solicit the opinion of each participating educator on the dynamics of the session - Thank everyone for their participation by emphasizing the importance of their opinion |

Annex 2 – Sociodemographic questionnaire


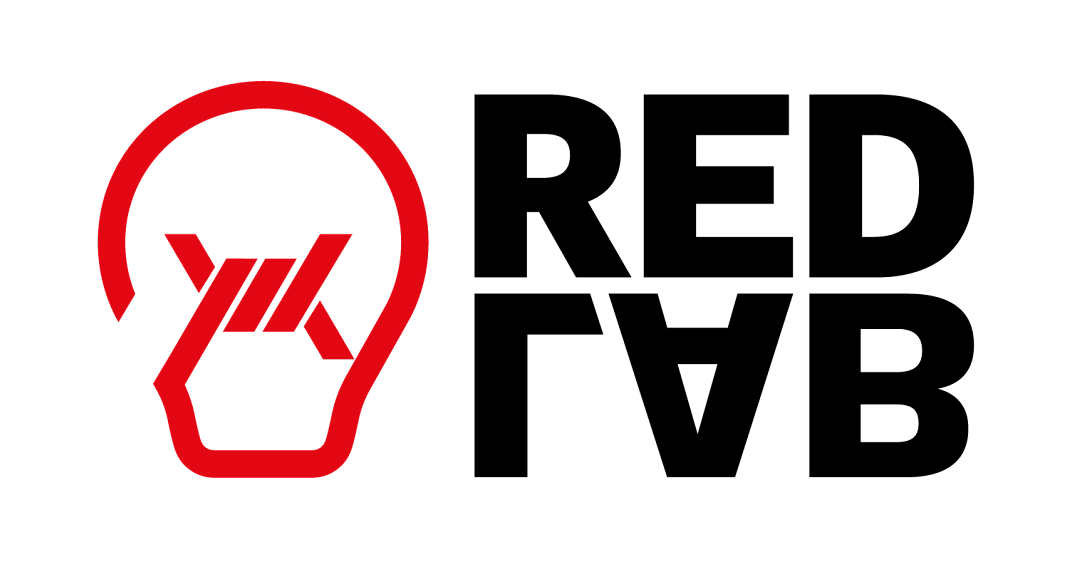


**RedLab – Info form**

Thankyou for participating in our activities! Please complete the form below with the information you agree to share with us.

1. First Name:

____________________________

1. Gender/sex:

____________________________

1. Age:

____________________________

1. Educational background:

____________________________

1. Do you attend a school or a training programme?

🞏 Yes

🞏 No

1. Do you have a job?

🞏 Yes

🞏 No

Data will be processed in compliance with the legislation in force concerning the protection of personal data.

The data collected in the survey will be treated with respect for privacy.


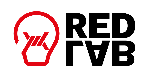


RedLab

Darkroom over the borders ETS

Via Emilio Saigari 2/A 37131 Verona (VR) CF 93289680238
